# Characterizing Dynein’s Role in P-cell Nuclear Migration using an Auxin-Induced Degradation System

**DOI:** 10.17912/W2W96J

**Published:** 2018-05-01

**Authors:** Jamie Ho, Venecia A. Valdez, Linda Ma, Daniel A. Starr

**Affiliations:** 1 Department of Molecular and Cellular Biology, University of California, Davis, CA 95616, USA

**Figure 1.**
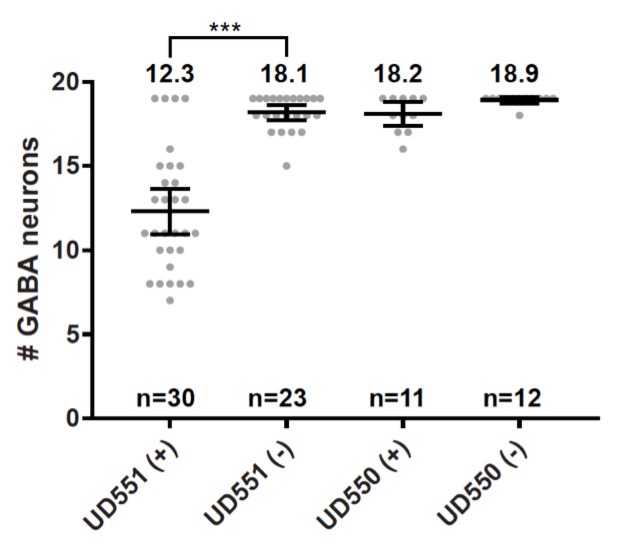
**Auxin-induced degradation of dynein causes a P-cell nuclear migration defect.** Average number of GABA neurons in L4 animals that express the TIR1 gene (an auxin-induced E3 ubiquitin ligase; Zhang et al., 2015) specifically in P cells. Animals either expressed the DHC-1 degron tag (UD551) or did not express the DHC-1 degron tag (UD550). These animals (UD551 and UD550) were either exposed to auxin (+) or not (-) as L1 animals. Statistical significance calculated by t-test with p-value<0.0001. The mean is marked with error bars denoting 95% CI.

## Description

Nuclear migration limits the rate of cellular migration through narrow spaces due to the large size and stiffness of the nucleus (Ungricht and Kutay, 2017). Using *Caenorhabditis elegans* as a model organism, we can observe P-cell nuclear migration *in vivo*. During the mid-L1 stage, P-cell nuclei that are about 3-4μm in diameter must migrate from a lateral to ventral position. This migration occurs through a constricted space ~ 200nm wide, about 5% of the diameter of the relaxed nucleus, between body wall muscle and cuticle (Cox and Hardin, 2004). If this migration succeeds, P-cells develop into vulval cells and GABA neurons. Failure of P-cell nuclear migration leads to cell death and missing P-cell lineages, leading to egg laying defective (Egl) and uncoordinated (Unc) animals because of missing vulval cells and GABA neurons, respectively (Sulston and Horvitz, 1981). Two proteins that are known to be involved in P-cell nuclear migration are UNC-84 and UNC-83. These proteins make up the LINC complex to form a bridge between the nucleus and the cytoplasm. Disruption of the LINC complex leads to nuclear migration defects in P-cells (Starr et al., 2001). Previously, our lab showed that P-cell nuclei migrate towards the minus ends of microtubules through the microtubule motor, dynein. Dynein is essential in embryogenesis (Gonczy et al., 1999), therefore our research was previously limited to viable, partial loss-of-function alleles of dynein or dynein-interacting proteins. Animals expressing a hypomorphic allele of dynein, *dhc-1(js319)*, had an average of ~3 P-cells that failed to migrate (Bone et al., 2016).

Here, we tested the hypothesis that the *unc-83/unc-84* pathway works through dynein to move P-cell nuclei using the auxin-inducible degradation system (AID) to knock down dynein specifically during P-cell nuclear migration (Zhang et al., 2015). The *Arabidopsis thaliana* TIR1 gene was expressed downstream of the *C. elegans* P-cell specific *hlh-3* promoter (Bone et al., 2016) in the strain UD550 (*oxIs12[unc-47::GFP]; ycEx253[phlh-3::TIR-1::mRuby; odr-1::rfp]*). Next, UD550 was crossed to a strain with *dhc-1(ie28[dhc-1::degron::GFP])* (Zhang et al., 2015) to make UD551 (*dhc-1(ie28[dhc-1::degron::GFP]) I; oxIs12[unc-47::GFP] X; ycEx253[phlh-3::TIR-1::mRuby; odr-1::rfp]*). UD550 and UD551 animals were synchronized to mid L1 as previously described (Bone et al., 2016) and exposed to 1mM auxin for five hours at 25°C. P-cell nuclear migration defects were quantified by counting GABA neurons marked with UNC-47::GFP by fluorescence microscopy in L4 animals after the auxin treatment. Wild-type animals have 19 GABA neurons and missing GABA neurons indicate that P-cell nuclear migration failed (Chang et al., 2013; Bone et al., 2016). As a negative control, UD550 animals, which lack the *dhc-1* degron tag, had no P-cell nuclear migration defects when exposed to auxin. This result is similar to UD551 animals that were not exposed to auxin (Figure 1). In support of our hypothesis, larvae exposed to auxin that expressed both the TIR1 gene in P cells and the *dhc-1* degron tag (UD551) had an average of 12.3 GABA neurons compared to sibling larvae not exposed to auxin that had an average of 18.1 GABA neurons (Figure 1). Thus, the dynein-degraded larvae had an average of 5.8 missing GABA neurons (p<0.0001), indicative of a severe P-cell nuclear migration defect. These results further strengthen our model that dynein plays a major role in generating forces to move nuclei in P-cells through constricted spaces. Finally, the *phlh-3::TIR1* line will be a valuable reagent to knock down other essential proteins to determine their roles during P-cell nuclear migration.

## Reagents

The *Arabidopsis thaliana* TIR1 gene was amplified from pLZ31 (pCFJ151_Peft-3_TIR1_linker_mRuby_unc-54 3’UTR; a gift from Abby Dernburg; Addgene plasmid # 71720; Zhang et al., 2015) and cloned downstream of the P-cell specific *hlh-3* promoter in pSL780 (Bone et al., 2016) to make pSL814. 2ng/ul of pSL814 and 100ng/ul of *odr-1::rfp* were injected into a strain with *oxIs12[unc-47::GFP]* to make the strain UD550: *oxIs12[unc-47::GFP]; ycEx253[phlh-3::TIR-1::mRuby; odr-1::rfp]*. UD550 was crossed to CA1207: *dhc-1(ie28[dhc-1::degron::GFP])*, a gift from Abby Dernburg (Zhang et al., 2015), to make UD551: *dhc-1(ie28[dhc-1::degron::GFP]) I; oxIs12[unc-47::GFP] X; ycEx253[phlh-3::TIR-1::mRuby; odr-1::rfp]*. Auxin (Sigma #I2886) was added to NGM plates to a final concentration of 1 mM.
